# Supplementary material for: Smoking and Vaping in Amateur Rugby Players, Coaches and Referees: Findings from a Regional Survey Might Help to Define Prevention Targets
Source: Int J Environ Res Public Health. 2021 May 26;18(11):5720. doi: 10.3390/ijerph18115720 (PMC8198015; doi:10.3390/ijerph18115720)
Supplement: Supplementary file 1 [file ijerph-18-05720-s001.zip › ijerph-1218167-supplementary.pdf]

Demographical data

I am : Male ◯ Female ◯

I am (year):

12	22	32	42
13	23	33	43
14	24	34	44
15	25	35	45
16	26	36	46
17	27	37	47
18	28	38	48
19	29	39	49
20	30	40	≥ 50
21	31	41	

I am (One or several answers)

Player ◯ Coach ◯ Referee ◯

My championship category is

"≤ 14" championship ◯ "> 14" championship ◯ Veteran championship ◯

Tobacco smoking and e-cigarette use as electronic nicotine delivery systems

Considering combustible cigarettes			Considering electronic cigarettes	
I am a daily smoker*			I am a daily vaper**	
I am a sometimes smoker*			I am a sometimes vaper**	
I have nearly never smoked			I have nearly never vaped	
I have never smoked			I have never vaped	
I have quitted ***			I have quitted ***	

*A daily or a sometimes smoker is defined by current smoking or stop smoking < 3 months
*A daily or a sometimes vaper is defined by current vaping or stop vaping < 3 months
*** Stop ≥ 3 months

Questions for daily or sometimes smokers

In average, how many combustible cigarettes do you smoke a day?

1 to 5	6 to 10	11 to 15	16 to 20	≥ 20

During the last 12 months, did you smoke ?

	Never	Sometimes	Often	Each time
Within 2h before training				
Within 2h after training				
Within 2h before a match				
Within 2h after a match				

Do you plan to quit?

YES NO

If yes, will you use electronic cigarette to help quitting?

YES NO

Question to former smokers

Did you quit smoking since at least one year?

YES NO

Questions for daily or sometimes vapers

During the last 12 months, did you vape?

	Never	Sometimes	Often	Each time
Within 2h before training				
Within 2h after training				
Within 2h before a match				
Within 2h after a match				

I use cartridges

With nicotine	Without nicotine	Both	I do not know

Do you plan to quit vaping?

YES NO

For what main reason did you start vaping?

As an aid to quit smoking		Because it is cheaper	
As an aid to avoid smoking		Because of curiosity	
To preserve performance		I prefer the taste	
Because it is less dangerous than smoking		To respect my neighbourhood	
To participate to a festive atmosphere		Other	

Question to former vapers

Did you quit vaping since at least one year?

YES NO

Nicotine products other than combustible cigarettes or electronic cigarettes

Do you use such products? YES NO

If YES,

Hookah		Nicotine patches	
Snus		Nicotine chewing gums	
Snuff		Other	
Cigars / cigarillos			

Questions to each respondent

For a person of your age, do you think that smoking is harmful?

Not at all	Moderately	Yes, of course	I do not know

Do you think it is more harmful to smoke in the 2 hours before sport than at rest?

Not at all	Moderately	Yes, of course	I do not know

Do you think it is more harmful to smoke in the 2 hours after sport than at rest?

Not at all	Moderately	Yes, of course	I do not know

Compared to nicotine contained in a combustible cigarette, do you think that nicotine contained in an electronic cigarette is?

Less harmful	As harmful	More harmful	I do not know

Compared to electronic cigarette cartridges containing nicotine, do you think that using cartridges without nicotine is?

Less harmful	As harmful	More harmful	I do not know

Do you think it is more harmful to vape in the 2 hours before sport than at rest?

Not at all	Moderately	Yes, of course	I do not know

Do you think it is more harmful to vape in the 2 hours before sport than at rest?

Not at all	Moderately	Yes, of course	I do not know
